# A Multicenter, Prospective Study of a New Fully Covered Expandable Metal Biliary Stent for the Palliative Treatment of Malignant Bile Duct Obstruction

**DOI:** 10.1155/2013/642428

**Published:** 2013-03-27

**Authors:** Bret T. Petersen, Michel Kahaleh, Richard A. Kozarek, David Loren, Kapil Gupta, Thomas Kowalski, Martin Freeman, Yang K. Chen, Malcolm S. Branch, Steven Edmundowicz, Michael Gluck, Kenneth Binmoeller, Todd H. Baron, Raj J. Shah, Timothy Kinney, William Ross, Paul Jowell, David Carr-Locke

**Affiliations:** ^1^Charlton 8, GI Endoscopy, Mayo Clinic, 200 1st Street SW, Rochester, MN 55905, USA; ^2^Weill Cornell Medical College, 1305 York Avenue, 4th Floor, New York, NY 10065, USA; ^3^University of Virginia, Charlottesville, VA, USA; ^4^Virginia Mason Seattle Main Clinic, 1100 Ninth Avenue, Seattle, WA 98101, USA; ^5^Thomas Jefferson University, Main Building, Suite 480, 132 South 10th Street, Philadelphia, PA 19107, USA; ^6^Cedars-Sinai Medical Center, 8700 Beverly Bl No. 7511, Los Angeles, CA 90048, USA; ^7^Hennepin County Medical Center, Minneapolis, MN, USA; ^8^GI Division, Department of Medicine, Hennepin County Medical Center, MMC 36, 420 Delaware Street SE, Minneapolis, MN 55455, USA; ^9^University of Colorado Boulder, Campus Box F735, Anschutz Inpatient Pavilion (AIP), S/M Gastroenterology, Therapeutic Endoscopy, USA; ^10^Duke University, DUMC 3662, Durham, NC 27710, USA; ^11^Washington University, School of Medicine, 660 South Euclid Avenue, Campus Box 8124, St. Louis, MO 63110, USA; ^12^California Pacific Medical Center, 2351 Clay Street No. 304, San Francisco, CA 94115, USA; ^13^University of Colorado, Campus Box F735, Anschutz Outpatient Pavilion (AOP), Room 2136-A, P.O. Box 6510, Aurora, CO 80045, USA; ^14^Hennepin County Medical Center, Medicine Office, GI 865B, 701 Park Avenue, Minneapolis, MN 55415, USA; ^15^The University of Texas MD Anderson Cancer Center, 1400 Pressler, Unit Number: Unit 1466, Houston, TX 77030, USA; ^16^Beth Israel Medical Center, 10 Union Square East, Suite 2G, New York, NY 10003, USA; ^17^Brigham and Women's Hospital, Boston, MA 02215, USA

## Abstract

*Background and Study Aims*. Endoscopic placement of self-expanding metal stents (SEMSs) is indicated for palliation of inoperable malignant biliary obstruction. A fully covered biliary SEMS (WallFlex Biliary RX Boston Scientific, Natick, USA) was assessed for palliation of extrahepatic malignant biliary obstruction. *Patients and Methods*. 58 patients were included in this prospective, multicenter series conducted under an FDA-approved IDE. Main outcome measurements included (1) absence of stent occlusion within six months or until death, whichever occurred first and (2) technical success, need for reintervention, bilirubin levels, stent patency, time to stent occlusion, and adverse events. *Results*. Technical success was achieved in 98% (57/58), with demonstrated acute removability in two patients. Adequate clinical palliation until completion of followup was achievedin 98% (54/55) of *evaluable* patients, with 1 reintervention due to stent obstruction after 142 days. Mean total bilirubin decreased from 8.9 mg/dL to 1.2 mg/dL at 1 month. Device-related adverse events were limited and included 2 cases of cholecystitis. One stent migrated following radiation therapy. *Conclusions*. The WallFlex Biliary fully covered stent yielded technically successful placement with uncomplicated acute removal where required, appropriate reduction in bilirubin levels, and low rates of stent migration and occlusion. This SEMS allows successful palliation of malignant extrahepatic biliary obstruction.

## 1. Introduction

Endoscopic stent placement is now a standard therapy for management of most biliary strictures [1–3]. Plastic stents are generally employed in patients with benign or indeterminate strictures and for malignant strictures in patients with limited life expectancy [4]. Self-expanding metal stents (SEMSs) are indicated in patients with confirmed inoperable malignant biliary strictures. Bare SEMS are typically employed at the porta hepatis, while bare, partially covered, or fully covered SEMS may be used for extrahepatic lesions. Bare and partially covered SEMSs are prone to late occlusion due to ingrowth through the struts of the stent or overgrowth at the ends by tumor or hyperplastic tissue [5]. Some designs of partially and fully covered SEMS may be more prone to migration [6]. A fully covered stent with design features that limit migration yet facilitate repositioning or removal would provide optimal palliation of malignant strictures without significant risk related to inaccurate placement, size selection, or ultimate tissue diagnosis. The WallFlex Biliary RX Fully covered Stent is designed to meet these needs. This study was undertaken to evaluate the safety and clinical effectiveness of this new stent for palliative treatment in patients with inoperable extrahepatic malignant biliary obstruction. 

## 2. Patients/Materials and Methods

This multisite, single arm, prospective study was performed at 10 centers under an Investigational Device Exemption (IDE) from the Food and Drug Administration (FDA). The study protocol and amendments were approved by Institutional Review Boards at all participating centers. All patients, or their legal representatives, provided written Informed Consent prior to enrollment. An Independent Medical Reviewer assessed events of stent occlusion or migration and all events resulting in death. This expert endoscopist was not an employee of the sponsor or involved with a participating center.

*Inclusion criteria* required (1) presence of inoperable malignant nonhilar extrahepatic biliary obstruction, (2) clinical symptoms of biliary obstruction, (3) age 18 or older, and (4) Willingness and ability to comply with study procedures and provide signed informed consent. *Exclusion criteria* included any of the following: (1) participation in another investigational study within 90 days prior to consent, (2) Strictures that could not be traversed by the delivery system, (3) perforation of any duct within the biliary tree, (4) presence of a biliary SEMS, (5) presence of any esophageal or duodenal stent, (6) contraindications to endoscopy, (7) sensitivity to any components of the stent or delivery system, (8) Active hepatitis, (9) intrahepatic metastases that extensively involve both lobes of the liver, and (10) life expectancy of <3 months.

The *primary endpoint* was adequate clinical palliation of biliary obstruction, as demonstrated by maintenance of bilirubin below 3 mg/dL or reduction by >30% if the baseline value was greater than 3 mg/dL and absence of evidence of stent occlusion until death or 6 months of followup, whichever occurred first. A stent was considered occluded if the patient presented with elevation of total bilirubin that the treating physician deemed to be due to recurrent biliary obstruction and endoscopic imaging evidence of obstruction. 

*Secondary endpoints* included (1) ability to deploy the stent in satisfactory position, (2) ability to successfully reposition or remove the stent, if required, at initial placement or at stent failure, without clinically significant complications or technical difficulties, (3) occurrence and severity of device and procedure-related adverse events, (4) need for reintervention, (5) reduction in symptoms of biliary obstruction (jaundice, pruritus, right upper quadrant pain, fever, nausea, vomiting, and dark urine) at all visits, (6) patency at 1, 3, and 6 months, and (7) time to stent occlusion.

### 2.1. Follow-Up Visits

Stent patency at 1 month was assessed by total bilirubin level and at 1, 3, and 6 months by lack of obstructive symptoms. Follow-up visits were primarily conducted by phone, with inperson unscheduled visits required in case of recurrent obstructive symptoms.

### 2.2. Enrollment and Analysis Cohorts

A total of 74 patients who signed the informed consent form (ICF) were considered *enrolled*. After enrollment, fourteen patients failed screening relative to eligibility criteria and were withdrawn from the study without study treatment. Two patients were successfully treated with the study device but later found not to have met eligibility criteria. Per protocol, these patients were not part of the *intent-to-treat (ITT) *cohort, defined as those patients who signed the ICF, met eligibility criteria, and had the stent placement procedure initiated. The *ITT *cohort of 58 patients was used for assessment of baseline information and safety. Per protocol, assessment of the primary endpoint and secondary effectiveness endpoints was performed on the *evaluable* cohort, defined as *ITT* patients with at least one week of followup. Three patients were excluded from the *evaluable* cohort on the basis of death unrelated to the device prior to 1 week of followup (2) or failed placement of the study device at the index procedure with conversion to another device (1). Hence, the *evaluable* cohort had 55 patients.

### 2.3. Stent Design

The Wallflex Biliary RX fully covered stent is a radiopaque SEMS made of braided nitinol with a translucent silicone polymer (Permalume) lining of its entire length to prevent tumor or hyperplastic tissue ingrowth, while maintaining friction between stent wires and bile duct wall. The stent ends are flared to minimize risk of migration. Looped wires at the ends aim to minimize the risk of impaling or ulcerating the mucosa. The stent has a retrieval loop at the distal end to facilitate removal or repositioning in the event of misplacement or migration. The flared ends collapse when subjected to a withdrawal force. The stent is available in 8 and 10 mm diameters of varying lengths (8 mm × 60 and 80 mm, 10 mm × 40, 60, and 80 mm).

The delivery system is a coaxial tube design. The outer sheath constrains the stent before deployment and can be advanced to reconstrain the stent, if repositioning is necessary, after partial deployment down to the marked reconstrainment limit (approx. 80% of the stent length).

### 2.4. Statistics

A literature review conducted prior to the study noted that the majority (~80%) of publications on covered biliary SEMS report stent occlusion rates of 25% or less. Therefore, for this study, 55 *evaluable* patients were required to test whether the stent occlusion rate at 6 months or death was significantly less than 25%, using a 1-sided binomial test with a significance level of 5% and a power of 90% for an expected stent occlusion rate of 10%.

Kaplan-Meier analysis was used to determine the rates of survival and stent occlusion based on available followup information, with censoring for discontinued and deceased patients and beyond six months after stent placement. The number of biliary obstructive symptoms at each followup was summarized by the mean and its 95% confidence interval, based on the normal distribution. A paired *t*-test was used to test the significance of reduction in the mean number of symptoms from baseline to 6 months, using the subset of patients with paired data available. Effectiveness measures were summarized by success rates and exact Clopper-Pearson 95% confidence intervals.

## 3. Results

The *ITT* cohort included 30 men and 28 women, with a mean age of 68.9 years (range 23–89.8). The top five reported biliary obstructive symptoms were jaundice (82.8%), dark urine (37.9%), right upper quadrant (RUQ) pain (34.5%), pruritus (32.8%), and nausea (17.2%). Tumor type and stricture location are summarized in Tables 1 and 2. Twenty patients (34.5%) had a previous biliary sphincterotomy.

### 3.1. Stent Placement and Removal

**Figure 1:**
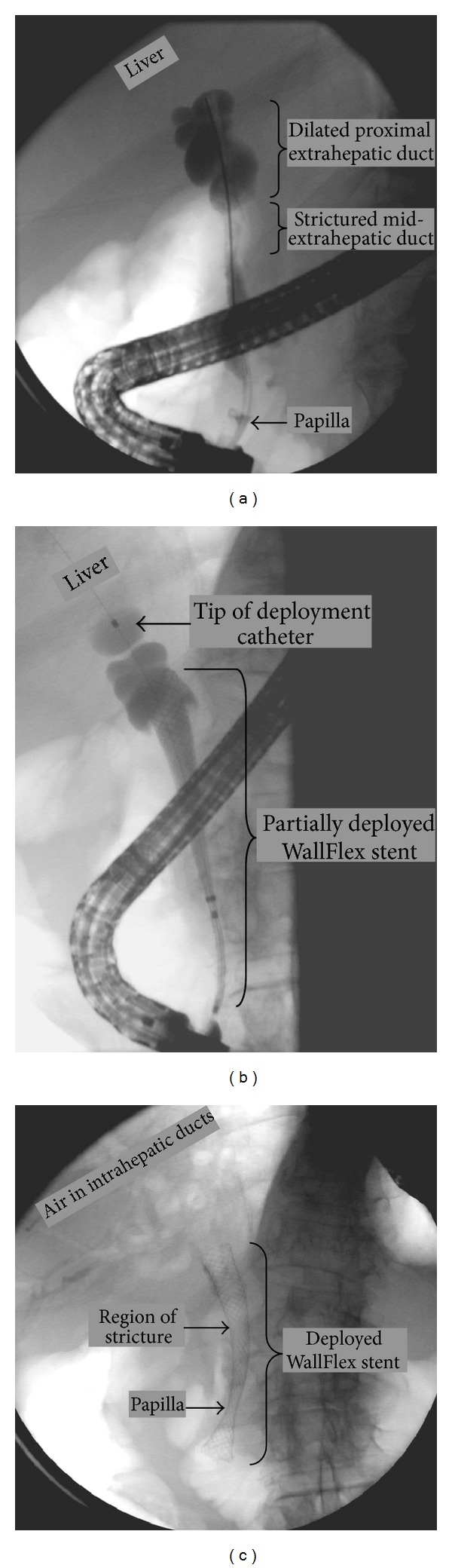
(a)–(c) Cholangiographic images of malignant biliary stricture and WallFlex Biliary Stent placement. (a) 254 × 254 mm (96 × 96 DPI), (b) 338 × 338 mm (72 × 72 DPI), and (c) 338 × 338 mm (72 × 72 DPI).

Stent placement (Figure 1) was technically successful in 57 of 58 patients (98%) in the *ITT* cohort (Table 3). Deployment was attempted but failed in 1 patient due to severe angulation of the duodenoscope. In this patient the deployment system was removed and a noninvestigational biliary SEMS was placed during the index procedure.

  Three stents were deployed but immediately removed from two patients to better match the pathologic anatomy. In one, a stent that was deemed to be of excessive length was deployed, but subsequently removed and replaced with a shorter sent. In the other patient, a 10 mm × 60 mm stent proved insufficiently long and a 10 mm × 40 mm stent placed through the initially placed stent did not expand completely within the tight stricture. Both were removed without technical or clinical complications, after which a 10 mm × 80 mm stent was placed in good position. Thus, three stents were successfully removed without adverse events (Table 3). No patients underwent attempted stent removal after the initial stent placement procedure. All 57 ultimately placed study stents were 10 mm in diameter, with lengths of 40 mm in 14%, 60 mm in 72%, and 80 mm in 14% of stents. 

### 3.2. Effectiveness

 A summary of key effectiveness results is provided in Table 3. Overall 54 of 55 *evaluable* patients (98%) had adequate clinical palliation of biliary obstruction until death or completion of followup. This rate of stent occlusion (2%) was significantly less than those commonly noted in the literature (25%) (*P* < 0.0001). Of the 55 patients, 23 were followed to 6 months, 25 died while on study, and 7 were discontinued prior to month 6 or death. One patient with discontinued followup presented with bacteremia, cholangitis, and fever due to stent occlusion 142 days after stent placement. In this case, the distal end of the stent was collapsed on itself at the level of the papilla with repositioning wire tines extending from the papilla which detached during stent manipulation. The distal end of the stent was balloon dilated, and a commercial uncovered SEMS was placed inside the occluded stent with prompt clinical improvement. Another patient had an ampullary cancer and adequate clinical palliation, but experienced asymptomatic stent migration at day 84, which was deemed a result of response to radiation therapy. The patient did not require restenting. Five additional patients were dropped from followup for reasons unrelated to the stent at 33, 45, 50, 138, and 161 days after placement, including 3 patients that underwent Whipple procedures despite not originally being considered surgical candidates, 1 patient categorized as lost to followup on day 138 despite multiple contact attempts after the week 1 visit when no biliary obstructive symptoms were reported, and 1 patient who withdrew consent early due to failing health.

Total bilirubin levels at baseline and month 1 were available for 45 of the 55 *evaluable* patients. Among them, 44/45 (97.8%) demonstrated successful maintenance of total bilirubin by the previously mentioned criteria, with the mean level reducing from 8.9 mg/dL (range 0.4–27.1) to 1.2 mg/dL (0.2–8.0). One patient with underlying hepatitis C and known cirrhosis failed to demonstrate successful reduction in total bilirubin at the month 1 visit (baseline total bilirubin = 11.0 mg/dL; month 1 = 8.0 mg/dL). In ten patients month 1 total bilirubin levels were not obtained due to early death (3) missing blood draw (3), early discontinuation (2), patient refusal (1), and loss to followup (1). 

The mean number of reported biliary obstructive symptoms was reduced at each visit compared to the prior visit, ranging from a baseline mean for all 55 evaluable patients of 2.73 ± 1.60 (standard deviation (SD)) (range 1–8, median 2) to 0.13 ± 0.46 (SD) reported symptoms (range 0–2, median 0) at the month 6 visit. Analyzing the subset of 23 subjects with paired data available, there was a significant reduction in the mean number of symptoms from baseline to 6 months (*P* < 0.0001). By protocol criteria, stent patency was evident in 91.3% at Month 6. 

**Figure 2:**
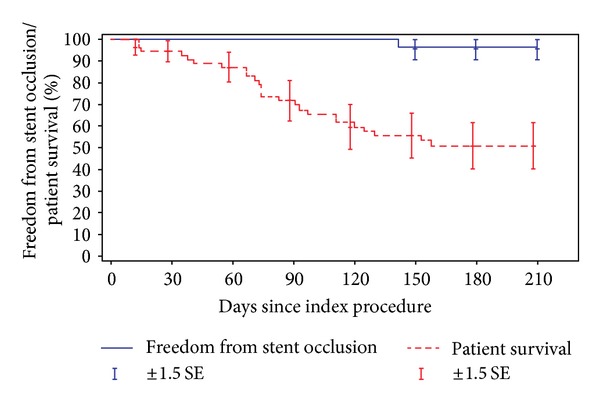
Kaplan-Meier curve of patient survival and freedom from stent occlusion 81 × 40 mm (300 × 300 DPI).

The estimated patient survival rate and the estimated rate of freedom from stent occlusion, as a function of time after stent placement, are both demonstrated in Kaplan Meier curves in Figure 2. One patient (1.8%) experienced a stent occlusion. Kaplan-Meier analysis based on the 55 evaluable patients yielded an estimated stent occlusion rate at 6 months of 3.7% (SD 3.6%). The median time to stent occlusion is at least 210 days. Hence, the study stent appears effective in providing biliary obstruction-free palliation to most patients until death or at least 6 months after stenting.

### 3.3. Safety

There were no unanticipated adverse device effects (UADEs), and no deaths were attributed to the investigational device. In the *ITT* cohort, 4 patients experienced 6 serious adverse events (SAE's) deemed potentially (3) or probably (3) related to the study device. Cholecystitis occurred in 2 patients, 1 of whom also experienced gallbladder perforation. In this patient, purulent bile and gallstones were noted at the time of prior plastic stent removal during the index procedure. In both patients who experienced cholecystitis the stents were placed across the cystic duct; however, it is unknown whether there was tumor involvement at that level. Both events resolved with antibiotics and percutaneous gallbladder drain placement. The prevalence of intact gallbladders among treated patients was not systematically documented; hence the rate of acute cholecystitis among those with intact gallbladders is unknown. Post-ERCP pancreatitis occurred in one patient who later also developed self-limited RUQ pain. One patient experienced severe, self-limited postprocedural nausea. The SAE of cholangitis/stent occlusion at day 142 noted in the effectiveness section was reported by the investigator as related to progression of underlying disease rather than the stent. The stent migration reported in the effectiveness section was not associated with an adverse event as it did not lead to patient symptoms. 

 Six additional patients experienced a total of 7 SAEs reported as probably or possibly related to the ERCP itself, including one case of respiratory compromise during procedural sedation, culminating in death 8 days later from resulting sequelae and underlying pancreatic cancer, and one case each of post-ERCP pancreatitis and of postsphincterotomy bleeding, both of which resolved without sequelae. Individual cases of fever, abdominal pain, and nausea that may have been procedure related resolved without sequelae.

## 4. Discussion

Studies of endoscopic palliation for malignant inoperable obstructive jaundice have demonstrated the therapeutic advantage and cost effectiveness of self-expanding metal stents compared to surgical bypass or placement of plastic stents [2, 14, 15]. The efficacy of bare metal stents is limited by the propensity for tumor ingrowth [16]. Similarly, partially covered SEMSs remain prone to obstruction by ingrowth and overgrowth of tumor and by hyperplastic tissue response at the short bare segment at the upper end. Thus, the potential benefit of the central covering is offset by the persistent risk of occlusion at the uncovered ends [12, 17–19]. Fully covered designs without sharp wire ends may preclude these complications and thereby yield prolonged freedom from stent occlusion. In this series, only one patient experienced recurrent biliary obstruction, after 142 days.

Recurrent biliary obstruction caused by migration or stent occlusion remains the most important issue. It is still unproven whether CSEMS will be a better solution than uncovered SEMS as evidenced by 2 recently published randomized studies, [9, 12]; however, a recent meta-analysis [20] showed significantly prolonged stent patency and stent survival for covered stents. Data from this series and Costamagna et al. [7] suggest that the covered WallFlex stent design may lead to a lower recurrent biliary obstruction rate compared to older designs (Table 4). Despite the full length covering on the WallFlex stent used in the present series, only one stent migrated, and this was asymptomatic in a patient with presumed stricture improvement following radiation therapy. Stent replacement was not required.

This study of a new fully covered SEMS demonstrates successful endoscopic palliation of unresectable malignant distal biliary obstruction in 98% of patients, suggesting that the goals of various stent design features were mostly met. As in most studies of biliary SEMS, technical placement success was almost universal. One stent failed to deploy adequately and was immediately replaced by a commercially available stent. Two patients' stents were incorrectly sized and therefore removed and replaced with different lengths. The narrow mesh design of the Wallflex stent foreshortens during placement and requires some attention to placement, compared to the wider mesh nonforeshortening laser cut designs.

 For practical purposes bare SEMSs are not removable following the procedure at which they were inserted. Although there are descriptions of successful removal of uncovered biliary stents, it is technically difficult to perform and not predictable [21]. The partially and fully covered metal stents do not carry labeling for removability in the USA; however, they have been employed “off-label” for limited durations in various nonmalignant conditions such as benign strictures [22, 23] and intractable bile leaks [23, 24]. The partially covered versions are still prone to proximal ingrowth and potentially difficult removal [25, 26]. This limits their use in benign applications, and some authors consider them inappropriate for use in patients with a life expectancy greater than 24 months [27]. In this study, we used a fully covered stent that is retained by collapsible flares at the ends, which make repositioning or immediate removal relatively atraumatic and highly predictable. In the porcine model this stent design was removed shortly after placement from all six animals without difficulty [28]. Similarly, in this study three stents were removed immediately without difficulty from two patients. If this ease of removal is confirmed in subsequent studies with longer indwell periods, this stent may alter the paradigm for palliation of indeterminate biliary strictures that are presumed to be malignant but are still undefined [21]. As reported by Siddiqui et al., [29] fully covered CSEMS can now be used as an initial intervention to relieve malignant biliary obstruction, even in patients whose surgical resectability status is uncertain; however, the use of the stent in such manner would be considered “off-label” in the USA. Placement prior to or at the time of biopsy and prior to assessment of operability could then proceed without concern about the risk for permanence in potentially benign lesions or in lesions likely to resolve with medical therapy, such as strictures related to lymphoma or IgG4-related disease. Of note in this series, three patients were originally considered inoperable but later went on to surgery and en bloc stent removal.

This study is limited by the lack of a comparison group. Also, 17 patients in this series died of their underlying disease within three months, limiting assessment of the duration of freedom from stent occlusion. Nevertheless, insertion success, duration of adequate clinical palliation, and complications were all superior or comparable to most published data on self-expanding biliary stents. Comparative trials of this fully covered stent versus noncovered or partially covered metal stents for extrahepatic malignant applications and versus multiple plastic stents for benign lesions are needed.

## Figures and Tables

**Table 1:** Types of malignancy at baseline.

Cancer type	ITT subjects (*N* = 58 subjects)
Pancreatic	45 (77.6%)
Cholangiocarcinoma	3 (5.2%)
Ampullary	2 (3.4%)
Colon	2 (3.4%)
Lung	2 (3.4%)
Other undefined malignancy	
Retroperitoneal mass, suspect sarcoma	
Malignant adenopathy of the porta hepatic portacaval, and pericardial regions	4 (6.9%)
Peripancreatic malignant lymphadenopathy	
Lymphoma to porta hepatis	

**Table 2:** Baseline stricture locations.

Location of stricture	Baseline *N* = 58 (%)
Distal common bile duct (CBD)	41 (70.7 %)
Mid CBD	9 (15.5%)
Proximal common bile duct	5 (8.6%)
Distal CBD and mid CBD	1 (1.7%)
Junction of distal CBD and mid CBD	1 (1.7%)
Papilla	1 (1.7%)

**Table 3:** Key effectiveness results.

Measure	Measure of success *N* (%)	[95% CI]
Primary endpoint		
Adequacy of palliation	54 (98.2%)	[90.3%, 100%]*
Secondary endpoints		
Stent deployment/technical success	57 (98.3%)	[90.8%, 100%]
Number of biliary obstructive symptoms at baseline		
Mean ± SD (*N*)	2.7 ± 1.76	[2.0, 3.50]
Range (Min, Max)	(1, 8)	
Median	2	
Number of biliary obstructive symptoms at month 6		
Mean ± SD (*N*)	0.1 ± 0.46	[0.00, 0.33]
Range (Min,Max)	(0, 2)	
Median	0	
Stent patency	21 (91.3%)	[72.0%, 98.9%]
Reduction of bilirubin level at month 1	44 (97.8%)	[88.2%, 99.9%]

*Intent-to-treat (58).

**Table 4:** Comparison to recent publications.

First author	Enrollment	Number of centers	Number of patients	Metal stent type and covering*	Recurrent biliary obstruction rate	Stent patency at 6 months	Migration rate	Cholecystitis rate	Pancreatitis rate
Costamagna [7]	5 months	6	66	PC WallFlex	6% (4)	94%	3% (2)	5% (3)	2% (1)

Isayama [8]	60 months	4	138	PC Wallstent	21% (29)	—	17% (24)	7% (9)	4% (5)
	11 months	20	141	PC WallFlex	26% (36)	78%	8% (11)	10% (14)	6% (8)

Kullman [9]	34 months	10	200	UC Nitinella	23% (45)	78%	0% (0)	1% (2)	2% (4)
			200	PC Nitinella	24% (47)	74%	3% (6)	1% (2)	3/200

Petersen	6 months	10	55	FC WallFlex	2% (1)	91%	2% (1)	4% (2)	2% (1)

Song [10]	19 months	1	24	Paclitaxel covered SEMS	21% (5)	—	0% (0)	0% (0)	4% (1)
			25	FC SEMS	32% (8)	—	16% (4)	0% (0)	0% (0)

Talreja [11]	41 months	6	260	FC WallFlex	3% (8)	63%	2% (4)	1% (2)	2% (4)

Telford [12]	68 months	4	61	UC Wallstent	18% (11)	90%	0% (0)	5% (3)	2% (1)
			68	PC Wallstent	29% (20)	87%	12% (8)	4% (3)	0% (0)

Tringali [13]	30 months	Unknown (multicenter)	70	PC ComVi	30% (21)	70%	4% (3)	1% (1)	0% (0)

*FC: fully covered; PC: partially covered; UC: uncovered.
